# Contextual variation in young children’s acquisition of social-emotional skills

**DOI:** 10.1371/journal.pone.0223056

**Published:** 2019-11-18

**Authors:** Dana C. McCoy, Jorge Cuartas, Marcus Waldman, Günther Fink

**Affiliations:** 1 Harvard Graduate School of Education, Cambridge, MA, United States of America; 2 Swiss Tropical and Public Health Institute and University of Basel, Basel, Switzerland; Pennsylvania State University, UNITED STATES

## Abstract

This study examined variation in the timing of 5,447 infants’ and toddlers’ reported acquisition of 12 basic social-emotional skills across and within 11 developing and developed country sites. Although children differed significantly across sites in when they attained social-emotional skills on average (e.g., *M* age Brazil = 20.50 months vs. *M* age India *=* 26.92 months), there was also substantial heterogeneity across skills. For example, children in Pakistan were reported to demonstrate sympathy on average seven months earlier than their peers in Ghana, whereas the opposite was true for sharing. Overall, country-level health and education were strongly associated (*r* > .60) with earlier site-level skill attainment. In addition to heterogeneity across sites, we also observed notable within-site variability in skill development (*ICC*s = .03 to .38). Future research is needed to identify sources of variability and how to promote skills that matter within a given context.

## Introduction

Bioecological and dynamic systems theories have long emphasized the importance of context for shaping children’s development over time [1,2] Research from cross-cultural and cultural psychology has repeatedly shown that parents’ caregiving strategies and expectations for their children’s development differ substantially across contexts [3,4,5], shaping the “cultural regularities”[6] and “developmental niches” [7] in which young children grow and learn. In turn, a number of studies have found contextual variation with regard to when and how children acquire basic developmental skills, ranging from sitting and walking [8,9] to counting [10] to object categorization and naming [11].

Despite strong theoretical and empirical support for the importance of context in shaping early child development, relatively little is known regarding the universality of social and emotional skills, specifically [12]. The successful acquisition of social-emotional competencies is a hallmark of the early childhood period [13]. During the first few years of life, children learn progressively to regulate their impulses, get along with others, understand and respond to emotions, focus their attention on salient stimuli, follow rules, and engage in culturally appropriate social interactions. In Western settings, the early development of these skills has been shown to predict individuals’ later-life outcomes, including school readiness and academic achievement, as well as adult health, earnings, and social behavior [14,15].

Only a handful of studies have explored variation in social and emotional skills across cultural contexts [16]. For example, in a study of elementary school-aged children [17], found notable differences in emotional appraisal of difficult situations across three cultures (Brahman, Tamang, and the United States), with children in the U.S. being more “problem focused and action oriented” than children from Nepal. The authors attributed these differences to a combination of cultural and socioeconomic factors, including values surrounding personal expression, group harmony, respect for authority, and caste structures. A study by House and colleagues [18] across six diverse sites found that children were less likely to demonstrate prosocial behavior over time in contexts where these acts were costly. Similarly, recent work by Munroe [19] found variation in three- to nine-year old’s levels of altruism across four small-scale societies (Belize, Kenya, Nepal, and American Samoa) that were directly correlated with the levels of collectivism demonstrated in each culture. In one of the few studies examining social-emotional development in very young children, Chen and colleagues [3] identified cross-cultural differences in toddlers’ patterns of behavioral inhibition, finding that Chinese children were more reticent to interact with mothers and strangers than their Canadian counterparts. Relatedly, Keller and colleagues [20] found differences in toddlers’ self‐recognition and self‐regulation across three different settings (Cameroonian Nso farmers and Greek and Costa Rican middle-class families). While acknowledging that dispositional factors may be at play, these authors largely attributed observed differences in the toddlers’ regulatory skills to differential socialization practices and parenting styles across the study contexts.

Importantly, the majority of work examining social-emotional development across cultural contexts has used small samples of preschool or school-aged children and has focused on comparisons using a relatively focused range of settings and skills. Rather than duplicating these efforts, the goal of the present paper is to build upon this body of research to explicitly compare the timing of infants’ and toddlers’ acquisition of 12 basic social-emotional skills spanning six domains (curiosity and imagination, obedience and respect, social competence and prosocial behavior, attention, behavioral self-regulation, and emotional competence) across and within 11 highly diverse international sites. We focus on these particular domains given their prevalence in existing frameworks of early social-emotional development [21,22,23], their conceptual breadth, as well as the fact that our own qualitative work has reinforced their relevance across a diverse range of cultural settings [24]. In addition to exploring these larger descriptive patterns, we also assess the extent to which sites’ median timing of skill acquisition varies based on country-level health, education, and socioeconomic characteristics. In doing so, we endeavor to provide the largest descriptive study of young children’s social-emotional development to date, complementing smaller-scale studies by identifying broader cross-cultural differences in patterns of skill development that can be further explained through future research using more nuanced methods and samples.

## Materials and methods

### Sample and procedure

Data for the present study come from the Caregiver Reported Early Development Instruments (CREDI) database, which includes information on the early development of approximately 15,000 infants and toddlers living in 17 high-, middle-, and low-income countries (see [24] for details). For the present analysis, we focus on a subset of 11 sites across 10 countries in which social-emotional data were available for the full age range of interest (0 to 35 months). This analytic sample includes 5,447 children, of whom 51.04 percent were male. The average age of children in this sample was 17.34 months (*SD* = 9.56; *range* = 0–35). Across sites, 26.04 percent of children’s caregivers had no education, 20.52 percent had completed primary school only, 23.79 percent had completed secondary school only, and 29.65 had completed some form of higher education.

Sites were selected for participation in the original CREDI database based on local interest (i.e., based on the voluntary participation of researchers conducting early childhood research in a given area). The overall sample is thus highly diverse and includes individuals from urban and rural settings, as well as a variety of socioeconomic backgrounds. For example, the proportion of caregivers with a tertiary education or higher ranged from 0.53% in Guatemala to 97.00% in India. Indeed, consistent with most prior cross-cultural work, none of the sites included in this study was nationally representative. As such, results must be interpreted at the level of the site, rather than at the level of the country. (See S1 Table for details of each site.)

The majority of individual sites (*n* = 8) used in-person interviews in which selected caregivers orally responded to CREDI items administered by a trained, local data collector. Data collectors were selected and trained by local research teams according to recommendations from the CREDI team. Specifically, data collectors were recommended to have a secondary school level of education, to be literate and fluent in the local languages, and to have experience conducting interviews with caregivers. Training consisted of a review of the CREDI items and protocols, as well as basic research best practices (e.g., how to ensure participant confidentiality, data quality, etc.). Three sites (in Brazil, India, and the U.S.) used national online surveys to gather data from literate caregivers. In total, five of the 11 sites were located in a lower middle-income country (Ghana, Guatemala, India, Pakistan, the Philippines), three were in a higher middle-income country (Brazil, Jordan, Lebanon), and three were in a high-income country (Chile, U.S.). Prior evidence from a sample of 68 U.S.-based caregivers suggests that CREDI scores collected using in-person interviews and online surveys are strongly correlated (*r*s > .65) [25]. Given this evidence, data from the U.S. sites were analyzed jointly for ease of interpretation.

The CREDI project represents a secondary data analytic effort using de-identified data. It was reviewed by the Harvard University Institutional Review Board and deemed human subjects exempt.

### Measures

#### Social-emotional skills

Children’s social-emotional skills were reported by caregivers using the CREDI. The CREDI is a caregiver-reported measure of 0- to 35-month-old children’s motor, language, cognitive, and social-emotional skills that was intentionally developed for cross-cultural, international use. A total of 149 CREDI items were developed to represent one or more of the major domains of early childhood development across cultural contexts (see [24] for details on the CREDI’s conceptual framework). Each item was also developed to be developmentally appropriate for the <36-month age period, easily reportable by caregivers, “culturally neutral” for global use, and psychometrically valid and reliable. Items were field tested in partnership with local child development experts across four pilot rounds using both qualitative and quantitative methods, including cognitive interviews assessing participant understanding and domain completeness, tests of reliability (e.g., test-retest reliability), and tests of validity (e.g., criterion validity with direct assessments). (For full item development details and validation results, see [24] .) For each CREDI item, caregivers were asked whether their child can or does exhibit a certain behavior and were instructed to respond by saying “yes” (1), “no” (0), or “don’t know” (missing).

The CREDI’s long form includes a social-emotional scale that is comprised of 23 items. Rather than using the full scale score (which would mask within-domain heterogeneity in specific skill attainment), the present analysis focuses on 12 items that were selected to reflect six core constructs of social-emotional development that have been found to be relevant for a variety of cultures and income levels (e.g., [26,27]). In particular, we selected two items in each of the following conceptual categories: (1) *curiosity and imagination* (“shows curiosity to learn new things” and “plays by pretending objects are something else”), (2) *obedience and respect* (“usually follows rules and obeys adults” and “greets neighbors or other people he/she knows without being told”), (3) *social competence and prosocial behavior* (“involves others in play” and “sometimes shares things with others without being told”), (4) *attention* (“can easily switch back and forth between activities” and “can concentrate on one task for 20 minutes”), (5) *behavioral self-regulation* (“often kicks, bites, or hits other children or adults” [reverse coded] and “frequently acts impulsively or without thinking” [reverse coded]), and (6) *emotional competence* (“shows sympathy or looks concerned when others are hurt or sad” and “can say what others like or dislike”). Each of the selected 12 items showed few (<10%) “don’t know” responses, were well understood by caregivers during cognitive interviews, and, with the exception of one item (“usually follows rules and obeys adults”), demonstrated adequate test-retest reliability over the course of 7 to 10 days (kappa of > .4; excepted item = .35). (For full item-level characteristics, see [24].) Differential item functioning (DIF) analysis showed evidence for measurement invariance of all 12 items across country income groups (i.e., high, middle, and low income countries). (For details of the DIF procedures and results, including item characteristic curves, see S1 Fig)

For analysis, the median age of attainment of each social-emotional skill was estimated within each site. (One exception was in the Philippines, where data on involving others in play were not available due to the local researchers choosing not to include this item in their study.) The full estimated distributions (logistic curves) of all social-emotional items in all sites are shown in S2 Fig.

#### Country characteristics

In the absence of site-level data on environmental features, country-level characteristics from the 2015 Human Development Index (HDI) database [28] were used to proxy the local health, educational, and economic conditions in which sample children were developing. These data include: life expectancy at birth (in years), expected years of schooling, gross national income (GNI) per capita in 2011 purchase power parity dollars (PPP$), and the HDI composite of these three variables. Descriptive statistics of these characteristics for the countries included in our analyses are shown in S2 Table.

## Results

### Timing of skill attainment across sites

**Fig 1:**
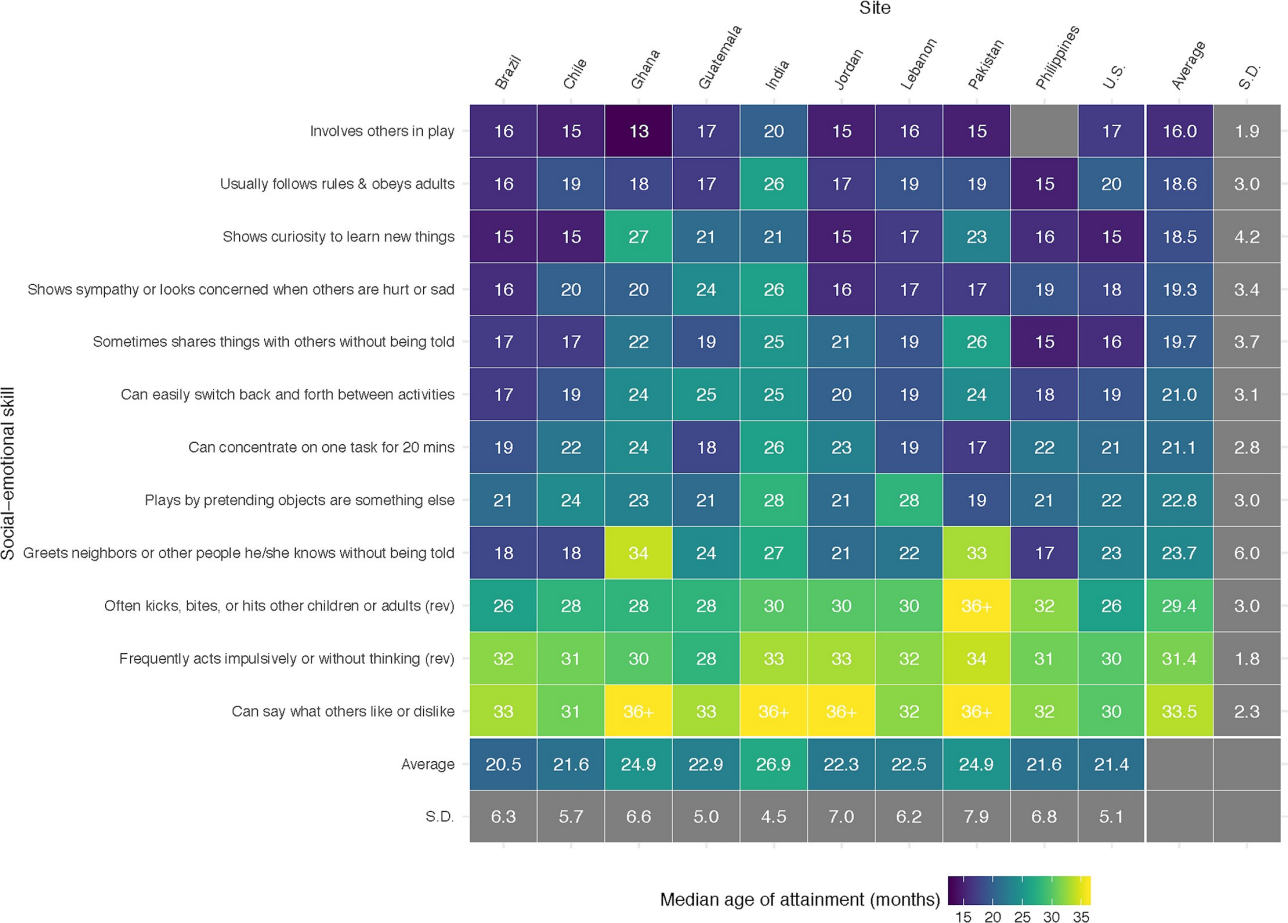
Median age (in months) of attainment of social-emotional milestones by site.

Fig 1 shows the median age (in months) at which children in different sites were reported to attain each of the 12 selected social-emotional skills, as well as the *SD* of these values across skills and sites. The site with the earliest median age of reported attainment across the 12 selected social-emotional skills was Brazil (*M* = 20.50 months; *SD* = 6.35), and the site with the latest median age of attainment was India (*M* = 26.92 months; *SD* = 5.02). Across sites, the earliest skill to develop was involving others in play (*M* = 16.00 months; *SD* = 1.94), whereas the latest to develop was the ability to say what others like or dislike (*M* = 33.50 months and *SD* = 2.32 across 6 sites; the remaining 4 sites did not demonstrate 50% completion by age 35 months and, as a result, a conservative value of 36 months was imputed for these four sites for the remainder of analyses).

Importantly, there was significant heterogeneity in the median age of each skill’s reported attainment across sites, as assessed by a joint significant test (*F*-test) of site fixed effects in a pooled logistic regression model predicting skill attainment as a function of age. As shown in S3 Table, the null of an equal age gradient across sites was strongly rejected for all 12 items analyzed (*p* < .001). Children in the Filipino site, for example, were reported by their caregivers to share objects with others by a median age of 15 months, whereas the average child in the Pakistani site was not reported to demonstrate this behavior until nearly a year later (26 months). The social-emotional skill with the least variation across sites was impulse control, which demonstrated a *SD* of 1.78 months across sites and a six-month median age difference between the earliest site (Guatemala; *M* = 28 months) and the latest site (Pakistan; *M* = 34 months). The skill with the most variation was greeting others, which showed a *SD* of 6.00 months across sites and ranged from a median age of attainment of 17 months in the Filipino site to 34 months in the Ghanaian site.

Although there was variability in the reported age of social-emotional skill attainment across sites, there was also heterogeneity in terms of which sites showed earlier versus later attainment, depending on the skill. The average rank-order correlation across pairs of skills was relatively low and non-significant at *r*_s_ = .26 (range = -.30 to .91; see S4 Table), indicating that sites where early development was reported for one skill did not always demonstrate early reported development for a different skill. For example, children in Ghana were earliest, on average, to involve others in play (*M* age = 13 months), but last to greet neighbors (*M* age = 34 months) and to express likes and dislikes (*M* age = >36 months). Similarly, children in Lebanon were relatively early to demonstrate sympathy (*M* = 17 months), but last (along with India) to engage in pretend play (*M* = 28 months). At the same time, a small set of skills showed very similar median age rankings across sites. For example, greeting neighbors and showing curiosity demonstrated a rank-order correlation across sites of .91, indicating that sites where children were reported to greet neighbors early tended to be the same settings where children were reported to show early curiosity, and vice versa.

### Associations between skill attainment and country-level characteristics

**Fig 2:**
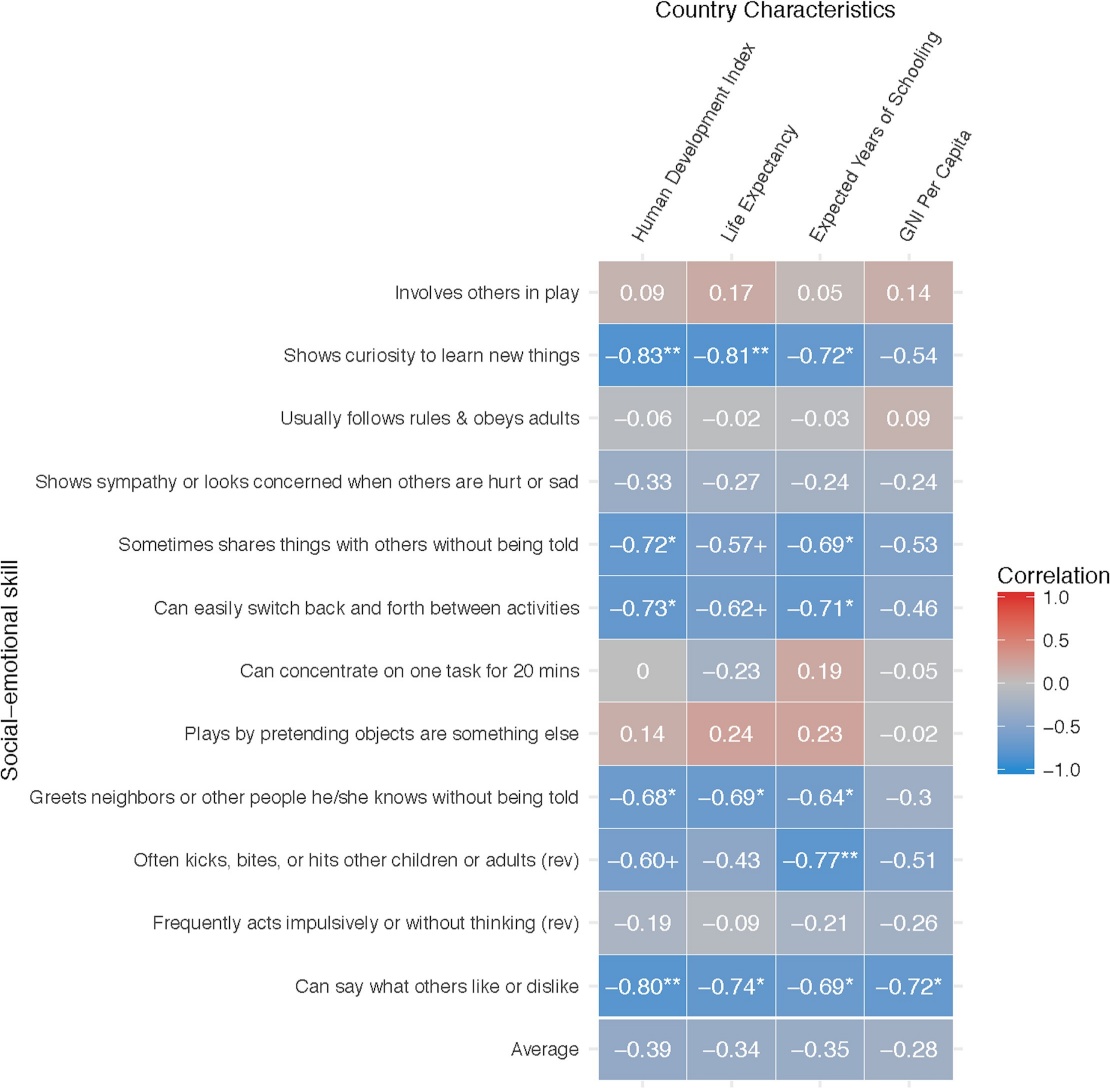
Correlations between site-level median age of skill attainment and country sociodemographic characteristics. All country characteristics taken from the 2015 Human Development Index database. Negative correlations imply that higher levels of country-level development are on average associated with earlier ages of skill acquisition.

Fig 2 highlights the correlations between sites’ median age of reported social-emotional skill attainment and country-level health, education, and socioeconomic wellbeing. Sites situated in countries with higher overall wellbeing–as represented by the HDI composite–showed significantly earlier curiosity (*r* = -.83, *p* < .01), identification of others’ likes/dislikes (*r* = -.80, *p* < .01), ability to switch back and forth between activities (*r* = -.73, *p* < .05), sharing without being told (*r* = -.72, *p* < .05), and ability to avoid aggressive behaviors like kicking, biting, and hitting (*r* = -.60, *p* < .10). These overall correlations appear to have largely been driven by the associations of site-level social-emotional skill development with country-level health (as represented by life expectancy) and education (as represented by expected years of schooling), rather than with socioeconomic status (as represented by per capita GNI). Importantly, however, country characteristics were not significantly associated with all social-emotional skills. The HDI, for example, was even shown to be weakly–and non-significantly–associated with *later* reported skill development in pretend play (*r* = .14, *p* = ns).

### Timing of skill attainment within sites

In addition to heterogeneity *across* sites in the timing of reported skill attainment, there was also notable variability *within* sites in terms of when children were reported to attain skills. Intra-class correlations (ICCs) were calculated using two-level logistic regression models predicting skill development controlling for age, with individuals nested in sites. These ICCs are shown in S3 Table and suggest that for all social-emotional skills, variation in the timing of reported skill acquisition was substantially larger within sites than it was across. Indeed, across skills, an average of 12 percent of variance was explained by site-level factors, with ICCs ranging from .03 for avoiding impulsivity to .28 for involving others in play. A look at the interquartile ranges (IQRs) of skill acquisition within sites confirms this variability descriptively (see S3 Table). For example, the mean IQR across sites for being able to switch back and forth between tasks was 13.43 months, suggesting that there was more than a year difference, on average, in the reported acquisition of this skill for children in the 25^th^ versus 75^th^ percentile. (See S1 Fig for site-specific logistic curves representing this variability for each skill.)

## Discussion

Despite the established importance of social-emotional skills and rising global interest in supporting them through intervention [29,30,31] (, relatively little is known about patterns of early social and emotional development across developing and developed countries. The results presented in this paper highlight substantial cross-contextual variability in the reported timing of infants’ and toddlers’ acquisition of a wide range of basic social-emotional skills. This variability manifests in several ways. First, we found variation in children’s social-emotional skill development across settings, with caregivers in some sites (e.g., Brazil, Chile, the Philippines, the U.S.) reporting earlier median skill attainment than others (e.g., Ghana, India, Pakistan). At the same time, we also found that the sites that showed early development in one skill did not necessarily demonstrate early development in another. For example, children in the Philippines were reported to demonstrate curiosity an average of almost a full year before their peers in Ghana, whereas Ghanaian children were reported to avoid aggressive behaviors approximately four months before their Filipino counterparts. Consistent with prior qualitative work [26] these results confirm substantial variability in the specific social-emotional competencies (e.g., curiosity, self-regulation, obedience and respect) that may be prioritized–and, in turn socialized–by a given culture or setting. They also highlight the importance of considering contextual variability in skills when developing and implementing social-emotional interventions [32].

Second, we observed variability in the degree to which a set of country-level contextual features predicted site-level social-emotional skill development. In general, we found earlier average skill development in countries experiencing more positive health, education, and socioeconomic conditions, supporting and extending a small but growing body of research on the effects of country-level adversity on young children’s outcomes [33]. At the same time, many social-emotional characteristics did not appear to be sensitive to these country-level conditions. Indeed, in the case of play-related behaviors, there was even a slight–though non-significant–trend toward later skill development in more advantaged contexts, suggesting that the broader conditions captured by the HDI cannot explain all variability in social-emotional skill development across contexts.

Third, in addition to variability across sites, we also observed large heterogeneity in reported skill development *within* sites. In the case of several skills–including sharing, switching between activities, concentrating, and greeting neighbors–there was on average more than a year of difference between when children in the 25^th^ versus 75^th^ percentile were reported to attain these skills. This finding confirms prior work from the U.S. showing substantial within-group variation in early childhood development outcomes [34,35], while also highlighting the limitations of solely focusing on group-level differences. Indeed, these findings emphasize the need to consider more individual and local sources of variability in social-emotional development *within* diverse contexts, including children’s temperamental characteristics, caregivers’ parenting and disciplinary practices, families’ socioeconomic wellbeing, and communities’ levels of resource availability [36,37,38,39]. Although we were unable to account for these characteristics in the present study due to a lack of data availability, doing so in future work will be critical for unpacking the mechanisms through which both local and country-level differences emerge.

Collectively, these results support prior narratives that social-emotional processes may be universal in nature–in that they exist in some form around the world–but that they are unlikely to be uniform in their timing, manifestation, and contextual relevance [40,41,42]. Indeed, rather than thinking of social-emotional development as a single domain with a common set of underlying predictors, this work suggests that social-emotional skills–even at their most basic–represent a vastly complex set of both related and unrelated constructs and ideas that are shaped by a variety of contextual and biological inputs. This conceptualization is supported by long-standing ecological and dynamic systems theories of human development that emphasize the importance of a wide array of bioecological characteristics for shaping young children’s behavior [1,43], as well as evidence from cultural and cross-cultural psychology demonstrating substantial variability in caregivers’ expectations and practices across settings [44].

One potential source of variation in children’s social-emotional skill development that was not explicitly captured in this study is the cultural tendency toward collectivism versus individualism. As noted above, this and other cultural preferences may serve as potential forces for shaping caregivers’ values (e.g., preference for duty, independence, etc.) and socialization practices (e.g., tendency to console versus minimize when children express negative emotions) and, in turn, children’s observable behavior [45,46,47,48]. At the same time, it is clear that this singular dimension of individualism-collectivism cannot explain all of the numerous differences observed in the present work. Indeed, our results suggest that children in India, Pakistan, and Ghana–three predominantly collectivistic cultures–were some of the last to demonstrate sharing behaviors, which have traditionally been associated with prosocial tendencies [49]. Prior large-scale research in adults has also raised questions about the extent to which individualism and collectivism can explain individual behavior. In their 2001 study of 23 international cities, for example, [50] found substantial variability in adults’ helping behaviors that did not appear to be associated with cultural characteristics of individualism and collectivism. Instead, much like the links between site-level social-emotional skill development and country-level health, education, and socioeconomic conditions observed in the present study, these authors found that adult helping behaviors were at least partially explained by broader economic and geographic factors, with Latin American city dwellers being more likely to help strangers than those in other parts of the world. Together, this work suggests that a variety of characteristics are likely to underlie the variation in skill development observed in the present study.

Overall, the results of this study can serve as a first step for exploring overall differences in young children’s development of a wide range of social-emotional skills across settings. Indeed, our overall conclusion that social-emotional skill attainment varies both within and across contexts is consistent with a host of research emphasizing the importance of multiple, interactive environmental and biological factors in shaping human development over time [1,5] . Although this study is to our knowledge the first to systematically describe a broad range of early social-emotional skills across highly heterogeneous country sites, additional research is needed to address this study’s limitations, including its non-representative, cross-sectional samples and relatively superficial measurement of complex social-emotional processes. Further measurement invariance analyses across and within sites are particularly needed to ensure that the cross-cultural comparisons being made in this and future work are valid. To improve the conceptual contributions of this work, future research using more comprehensive databases is needed to “unpack” the social, cultural, economic, and dispositional factors that may underlie the observed differences in this study. Furthermore, future work should consider building upon existing frameworks for conceptualizing social-emotional wellbeing to reflect local priorities and imperatives. Doing so will help to promote more effective, ecologically valid approaches to intervention that optimize the skills necessary for children’s success both locally and globally.

## Supporting information

S1 Table Site characteristics. (DOCX)

S2 Table Country characteristics. All country characteristics taken from the 2015 Human Development Index database.
(DOCX)

S3 Table Variability in timing of social-emotional skill development across and within sites. *F*-statistics and *p*-values are based on a joint significance test of site fixed effects in a pooled logistic regression model predicting skill development as a function of age. Intraclass correlations (ICCs) calculated using two-level logistic regression models predicting skill development controlling for age, with individuals nested in sites. 25% represents the average age at which 25 percent of children within a site attained the skill, 75% represents the average age at which 75 percent of children within a site attained the skill, and the interquartile range (IQR) represents the difference between the 75^th^ and 25^th^ percentile.
(DOCX)

S4 Table Rank-order correlations between median age of attainment of social-emotional skills across sites. + *p* < .10; * *p* < .05; ** *p* < .01. Correlations based on 11 observations, each representing the median age of skill attainment within a given study site.
(DOCX)

S1 Fig DIF analysis using IRT to investigate invariance in measurement across country-income subgroups for items included in the study. (DOCX)

S2 Fig Logistic curves showing proportion of children achieving a particular social-emotional skill across sites by month. (DOCX)

S1 Data Data file. (DTA)
