# Supplementary material for: Contextual variation in young children’s acquisition of social-emotional skills
Source: PLoS One. 2019 Nov 18;14(11):e0223056. doi: 10.1371/journal.pone.0223056 (PMC6860446; doi:10.1371/journal.pone.0223056)
Supplement: S1 Table — (DOCX) [file pone.0223056.s001.docx]

**Supporting Information Table 1.** Site characteristics

| **Country** | **Format** | ***N*** | **Caregiver Education** | | | | **Child Age** | | **Child % Male** | **Site Information** | |
| --- | --- | --- | --- | --- | --- | --- | --- | --- | --- | --- | --- |
|  |  |  | **None** | **Primary** | **Secondary** | **Tertiary** | ***M*** | ***SD*** |  | **Location** | **Description** |
| Brazil | Online | 624 | 0.81 | 1.79 | 30.03 | 67.37 | 21.08 | 8.54 | 54.98 | National | Geographically diverse children from the five regions of Brazil (majority from the southeast region) recruited through Facebook group of local child health television program followers. |
| Chile | Interview | 244 | 0.00 | 15.16 | 69.26 | 15.57 | 17.62 | 10.13 | 50.41 | Santiago | Socioeconomically diverse children from urban areas in the nation's capital. |
| Ghana | Interview | 1,539 | 71.09 | 17.58 | 10.03 | 1.30 | 16.61 | 9.55 | 48.93 | Northern Region | Representative random sample of six districts in Northern Ghana. All families were enrolled into a behavior change study. Primary source of income is farming; most households below international poverty line. |
| Guatemala | Interview | 189 | 19.05 | 58.73 | 21.69 | 0.53 | 16.61 | 9.36 | 53.51 | San Juan Ostuncalco & Quetzaltenango | Socioeconomically diverse rural and peri-urban children recruited through a parenting intervention. |
| India | Online | 200 | 1.00 | 0.50 | 1.50 | 97.00 | 18.33 | 10.56 | 51.00 | National | Socioeconomically advantaged and geographically diverse children recruited through Amazon Mechanical Turk. |
| Jordan | Interview | 341 | 21.47 | 57.94 | 16.47 | 4.12 | 16.13 | 9.56 | 51.03 | Sweileh, Marqa, & Mafraq | Jordanian and refugee (Syrian, Iraqi, and Palestinian) children from low-income urban settings recruited through NGO serving vulnerable populations. |
| Lebanon | Interview | 451 | 12.03 | 64.59 | 10.02 | 13.36 | 15.10 | 9.99 | 52.11 | Tripoli, Bekaa, Beirut, Saida, & Tyre | Lebanese and refugee (Syrian and Palestinian) children from socioeconomically diverse, urban and rural settings recruited from NGOs and local private nurseries. |
| Pakistan | Interview | 241 | 57.85 | 24.22 | 15.25 | 2.69 | 18.04 | 10.13 | 49.38 | Naushero Feroze, Sindh province | Predominantly low-income, rural and peri-urban younger siblings of children participating in a research study evaluating an ECD program. |
| Philippines | Interview | 719 | 0.83 | 16.55 | 60.78 | 21.84 | 18.03 | 7.84 | 51.46 | South Central Mindanao & Luzon | Socioeconomically diverse children from rural, peri-urban, and urban settings who were participating in an evaluation of programming through an NGO. |
| U.S. | Interview | 154 | 0.00 | 0.00 | 0.00 | 100.00 | 16.28 | 9.67 | 47.40 | Boston | High-SES, urban children recruited primarily through child care centers and pediatric clinics in the Boston region. |
| U.S. | Online | 745 | 1.75 | 2.82 | 22.04 | 73.39 | 16.76 | 10.11 | 51.95 | National | Socioeconomically and geographically diverse children recruited through Amazon Mechanical Turk. |
| *Total* |  | *5,447* | *26.04* | *20.52* | *23.79* | *29.65* | *17.34* | *9.56* | *51.04* |  |  |
