# Supplementary material for: Contextual variation in young children’s acquisition of social-emotional skills
Source: PLoS One. 2019 Nov 18;14(11):e0223056. doi: 10.1371/journal.pone.0223056 (PMC6860446; doi:10.1371/journal.pone.0223056)
Supplement: S2 Table — All country characteristics taken from the 2015 Human Development Index database. (DOCX) [file pone.0223056.s002.docx]

**Supporting Information Table 2***.* Country characteristics

|  | **Human Development Index (HDI)** | **Life Expectancy at Birth (yrs)** | **Expected Years of Schooling (yrs)** | **Gross national income (GNI) per capita (2011 PPP$)** |
| --- | --- | --- | --- | --- |
| Brazil | 0.754 | 74.7 | 15.2 | 14,145 |
| Chile | 0.847 | 82.0 | 16.3 | 21,665 |
| Ghana | 0.579 | 61.5 | 11.5 | 3,839 |
| Guatemala | 0.640 | 72.1 | 10.7 | 7,063 |
| India | 0.624 | 68.3 | 11.7 | 5,663 |
| Jordan | 0.741 | 74.2 | 13.1 | 10,111 |
| Lebanon | 0.763 | 79.5 | 13.3 | 13,312 |
| Pakistan | 0.550 | 66.4 | 8.1 | 5,031 |
| Philippines | 0.682 | 68.3 | 11.7 | 8,395 |
| U.S. | 0.920 | 79.2 | 16.5 | 53,245 |
| *Sample Average* | *0.710* | *72.6* | *12.8* | *14,247* |
| *Global Average* | *0.717* | *71.6* | *12.3* | *14,447* |

*Note*: All country characteristics taken from the 2015 Human Development Index database.
