# Supplementary material for: Contextual variation in young children’s acquisition of social-emotional skills
Source: PLoS One. 2019 Nov 18;14(11):e0223056. doi: 10.1371/journal.pone.0223056 (PMC6860446; doi:10.1371/journal.pone.0223056)
Supplement: S3 Table — F-statistics and p-values are based on a joint significance test of site fixed effects in a pooled logistic regression model predicting skill development as a function of age. Intraclass correlations (ICCs) calculated using two-level logistic regression models predicting skill development controlling for age, with individuals nested in sites. 25% represents the average age at which 25 percent of children within a site attained the skill, 75% represents the average age at which 75 percent of children within a site attained the skill, and the interquartile range (IQR) represents the difference between the 75th and 25th percentile. (DOCX) [file pone.0223056.s003.docx]

**Supporting Information Table 3.** Variability in timing of social-emotional skill development across and within sites

|  | ***Across Site Variability*** | | ***Within Site Variability*** | | | |
| --- | --- | --- | --- | --- | --- | --- |
|  | ***F_1,9_*** | ***p-value*** | ***ICC*** | ***25%*** | ***75%*** | ***IQR*** |
| Involves others in play | 532.70 | <.000 | 0.28 | 12.0 | 20.1 | 8.2 |
| Shows curiosity to learn new things | 439.00 | <.000 | 0.15 | 13.2 | 23.4 | 10.2 |
| Usually follows rules & obeys adults | 125.89 | <.000 | 0.08 | 13.2 | 24.1 | 10.9 |
| Shows sympathy or looks concerned when others are hurt or sad | 132.91 | <.000 | 0.11 | 14.0 | 24.4 | 10.4 |
| Sometimes shares things with others without being told | 184.53 | <.000 | 0.10 | 13.7 | 25.9 | 12.2 |
| Can easily switch back and forth between activities | 142.11 | <.000 | 0.06 | 14.7 | 28.2 | 13.4 |
| Can concentrate on one task for 20 mins | 78.94 | <.000 | 0.04 | 14.5 | 27.7 | 13.2 |
| Plays by pretending objects are something else | 84.32 | <.000 | 0.06 | 16.9 | 28.8 | 11.9 |
| Greets neighbors or other people he/she knows without being told | 407.12 | <.000 | 0.17 | 16.8 | 29.5 | 12.7 |
| Often kicks, bites, or hits other children or adults (rev) | 171.81 | <.000 | 0.17 | 25.8 | 32.4 | 6.6 |
| Frequently acts impulsively or without thinking (rev) | 32.88 | <.000 | 0.03 | 27.4 | 34.9 | 7.5 |
| Can say what others like or dislike | 105.23 | <.000 | 0.14 | 30.2 | 35.7 | 5.5 |
| *Average* | *203.12* | *<.000* | *0.12* | *17.7* | *27.9* | *10.2* |

*Notes*: *F*-statistics and *p*-values are based on a joint significance test of site fixed effects in a pooled logistic regression model predicting skill development as a function of age. Intraclass correlations (ICCs) calculated using two-level logistic regression models predicting skill development controlling for age, with individuals nested in sites. 25% represents the average age at which 25 percent of children within a site attained the skill, 75% represents the average age at which 75 percent of children within a site attained the skill, and the interquartile range (IQR) represents the difference between the 75^th^ and 25^th^ percentile.
