# Supplementary material for: Contextual variation in young children’s acquisition of social-emotional skills
Source: PLoS One. 2019 Nov 18;14(11):e0223056. doi: 10.1371/journal.pone.0223056 (PMC6860446; doi:10.1371/journal.pone.0223056)
Supplement: S4 Table — + p < .10; * p < .05; ** p < .01. Correlations based on 11 observations, each representing the median age of skill attainment within a given study site. (DOCX) [file pone.0223056.s004.docx]

**Supporting Information Table 4.** Rank-order correlations between median age of attainment of social-emotional skills across sites

|  |  | (1) | (2) | (3) | (4) | (5) | (6) | (7) | (8) | (9) | (10) | (11) |
| --- | --- | --- | --- | --- | --- | --- | --- | --- | --- | --- | --- | --- |
| (1) | Involves others in play |  |  |  |  |  |  |  |  |  |  |  |
| (2) | Shows curiosity to learn new things | -0.17 |  |  |  |  |  |  |  |  |  |  |
| (3) | Usually follows rules & obeys adults | 0.36 | 0.13 |  |  |  |  |  |  |  |  |  |
| (4) | Shows sympathy or looks concerned when others are hurt or sad | 0.38 | 0.46 | 0.49 |  |  |  |  |  |  |  |  |
| (5) | Sometimes shares things with others without being told | -0.24 | 0.77* | 0.13 | 0.17 |  |  |  |  |  |  |  |
| (6) | Can easily switch back and forth between activities | 0.17 | 0.74* | 0.20 | 0.67* | 0.72* |  |  |  |  |  |  |
| (7) | Can concentrate on one task for 20 mins | -0.05 | -0.04 | 0.29 | 0.33 | 0.13 | 0.15 |  |  |  |  |  |
| (8) | Plays by pretending objects are something else | 0.24 | -0.01 | 0.57 | 0.47 | -0.11 | -0.04 | 0.59+ |  |  |  |  |
| (9) | Greets neighbors or other people he/she knows without being told | -0.05 | 0.91** | 0.32 | 0.48 | 0.70* | 0.76* | 0.07 | -0.04 |  |  |  |
| (10) | Often kicks, bites, or hits other children or adults (rev) | -0.18 | 0.45 | 0.27 | -0.03 | 0.82** | 0.48 | -0.04 | 0.00 | 0.36 |  |  |
| (11) | Frequently acts impulsively or without thinking (rev) | -0.12 | 0.02 | 0.18 | -0.42 | 0.58 | 0.02 | 0.02 | -0.14 | -0.01 | 0.73* |  |
| (12) | Can say what others like or dislike | -0.3 | 0.61+ | -0.18 | 0 | 0.90** | 0.62+ | 0.26 | -0.26 | 0.55 | 0.60+ | 0.53 |
| *Notes*: + *p* < .10; * *p* < .05; ** *p* < .01. Correlations based on 11 observations, each representing the median age of skill attainment within a given study site. | | | | | | | | | | | | |
