# Supplementary material for: Contextual variation in young children’s acquisition of social-emotional skills
Source: PLoS One. 2019 Nov 18;14(11):e0223056. doi: 10.1371/journal.pone.0223056 (PMC6860446; doi:10.1371/journal.pone.0223056)
Supplement: S1 Fig — (DOCX) [file pone.0223056.s005.docx]

We used item response theory (IRT) methods to estimate differential item functioning (DIF) of the 12 social-emotional items included in our study across different cultural settings. Given the relatively small sample size in each country and each group, we split the available data three country income subgroups (i.e., high, middle, and low income countries), as we had done in prior analyses using the CREDI (see McCoy et al., 2018). IRT offers several features that are useful to analyze DIF. First, in contrast to classical test theory, IRT estimates item characteristics that are assumed as invariant across populations and independent of other items embedded in the test (Brennan, National Council on Measurement in Education, & American Council on Education, 2006). Second, IRT estimates both item discrimination (i.e., the extent to which the item is capable of distinguishing between individuals with different levels of the latent trait) and item location (i.e., the level of the latent trait where the scale is most reliable and precise in distinguishing between individuals), which are parameters that can be used to assessed DIF. Finally, IRT recognizes that the scale will be more precise at distinguishing between individuals in specific segments of the latent continuum, so it will be more imprecise at the extremes of the construct (Embretson & Reise, 2013).

Our IRT DIF analysis followed a standard series of steps. First, we defined a set of matching criterion or “anchor” items, external to the items to be assessed. For the present study, the following 10 items from the CREDI scale were used as anchors:

- Does the child recognize his/her name or nickname? That is, does he/she respond differently to his/her name than to other sounds or words?
- Does the child smile when others smile at him/her?
- Does the child often show affection toward others (e.g., hugging parents, brothers, or sisters)?
- Can the child indicate when he/she needs to go to the toilet?
- Does the child imitate others' behaviors (e.g., washing hands or dishes)?
- Can the child tell you when he/she is tired or hungry?
- Is the child kind to younger children (e.g., speaks to them nicely and touches them gently)?
- Does the child usually put objects or toys back where they belong after using them?
- Does the child sometimes save things like candy or new toys for the future?
- Does the child ask about familiar people other than parents when they are not there (e.g., "Where is the neighbor?")?

These items were chosen among those available from all countries to provide the largest possible range of difficulties.

Second, subgroup differences were analyzed conditional on the anchor items. In particular, we fit IRT models in Mplus 8.3 (Muthén & Muthén, 2017), fixing the mean and variance of anchor items and letting the parameters for the 12 items included in the study to be freely estimated across country income subgroups. Finally, subject matter experts assessed the extent to which any DIF identified in the study items was construct relevant or may have been indicative of bias.

Supporting Information Figure 1 presents the results of the DIF analyses for the 12 social-emotional items included in the study. The x-axis shows the level of the latent trait or “theta,” which in this case represents the level of social-emotional skill of the child. The y-axis presents the likelihood of answering the item correctly at each point of theta. As shown in the graph, item location and discrimination (as defined above) are strikingly similar across country income subgroups. What this suggests is that the level of theta (i.e., level of social-emotional skill) required for answering each item correctly is similar across income subgroups. This also suggests that the items are equally precise in differentiating children with different levels of theta across subgroups. Importantly, some items (see switching between activities, sharing, and greeting others in Supporting Information Figure 1) present small differences in the discrimination (as shown by the slope) at the extreme of the curve. However, considering the above discussion about IRT recognizing more imprecision at the extremes of the construct, this is a natural phenomenon that indicates fewer cases having such high levels of theta.

In sum, results from the DIF analysis strongly suggest that the items are invariant across income subgroups.

**Supporting Information Figure 1.** DIF analysis using IRT to investigate invariance in measurement across country-income subgroups for items included in the study


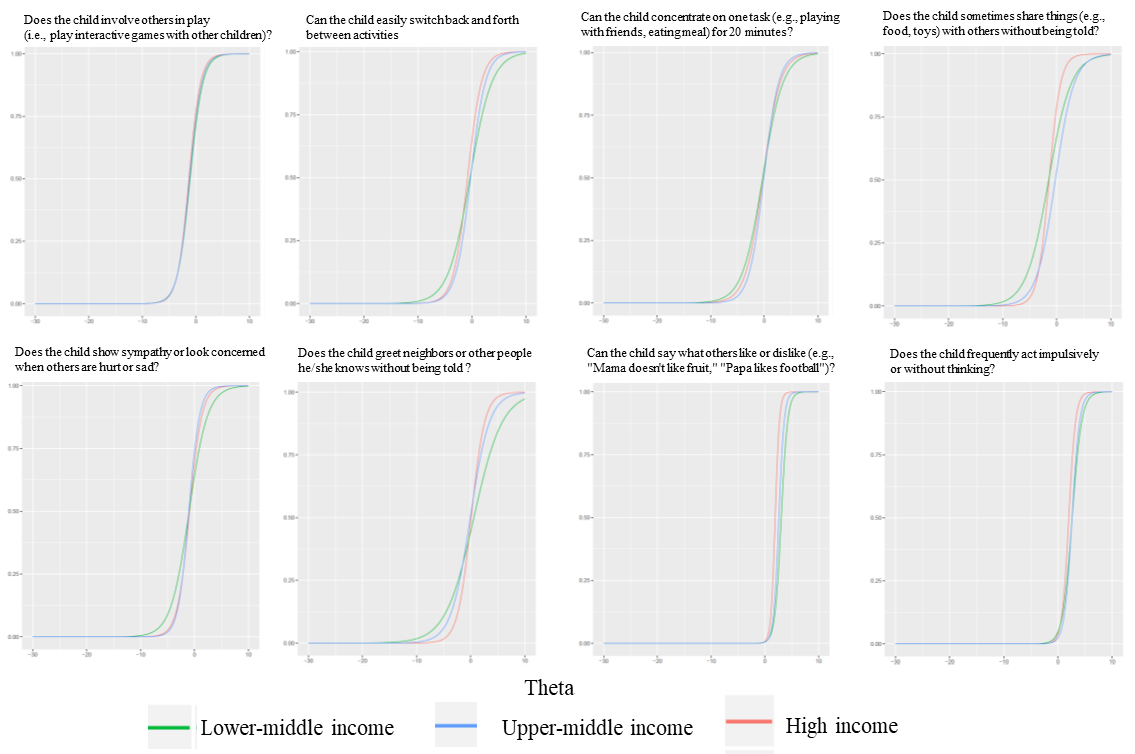


**References**

Brennan, R. L., National Council on Measurement in Education, & American Council on Education. (2006). *Educational measurement* (4th ed. ed.). Westport, CT.

Embretson, S. E., & Reise, S. P. (2013). *Item response theory*: Psychology Press.

Koretz, D. M. (2008). *Measuring up : what educational testing really tells us*. Cambridge, Mass.

Muthén, L. K., & Muthén, B. O. (2017). Mplus user's guide. Los Angeles, CA: Muthén & Muthén.
