# Supplementary material for: Contextual variation in young children’s acquisition of social-emotional skills
Source: PLoS One. 2019 Nov 18;14(11):e0223056. doi: 10.1371/journal.pone.0223056 (PMC6860446; doi:10.1371/journal.pone.0223056)
Supplement: S2 Fig — (DOCX) [file pone.0223056.s006.docx]

**Supporting Information Figure 1.** Logistic curves showing proportion of children achieving a particular social-emotional skill across sites by month

a. Involves others in play

b. Shows curiosity to learn new things

c. Usually follows rules and obeys adults

d. Shows sympathy or looks concerned when others are sad or hurt

e. Sometimes share things with others without being told

f. Can easily switch back and forth between activities

g. Can concentrate on one task for 20 minutes

b. Plays by pretending objects are something else

h. Greet neighbors or other people he/she knows without being told

i. Often kicks, bites, or hits other children or adults (rev)

j. Frequently acts impulsively or without thinking (rev)

k. Can say what others like or dislike
